# Pioneer farming in southeast Europe during the early sixth millennium BC: Climate-related adaptations in the exploitation of plants and animals

**DOI:** 10.1371/journal.pone.0197225

**Published:** 2018-05-18

**Authors:** Maria Ivanova, Bea De Cupere, Jonathan Ethier, Elena Marinova

**Affiliations:** 1 Institut für Ur-und Frühgeschichte und Vorderasiatische Archäologie, Universität Heidelberg, Heidelberg, Germany; 2 Royal Belgian Institute of Natural Sciences, Brussels, Belgium; 3 Landesamt für Denkmalpflege am Regierungspräsidium Stuttgart, Referat 84.1/ Archäobotanik, Gaienhofen-Hemmenhofen, Germany; 4 Laboratory of Biodiversity and Evolutionary Genomics, KU Leuven, Leuven, Belgium; University at Buffalo - The State University of New York, UNITED STATES

## Abstract

The Old World farming system arose in the semi-arid Mediterranean environments of southwest Asia. Pioneer farmers settling the interior of the Balkans by the early sixth millennium BC were among the first to introduce southwest Asian-style cultivation and herding into areas with increasingly continental temperate conditions. Previous research has shown that the bioarchaeological assemblages from early farming sites in southeast Europe vary in their proportions of plant and animal taxa, but the relationship between taxonomic variation and climate has remained poorly understood. To uncover associations between multiple species and environmental factors simultaneously, we explored a dataset including altitude, five bioclimatic and 30 bioarchaeological variables (plant and animal taxa) for 57 of the earliest farming sites in southeast Europe using Canonical Correspondence Analysis (CCA). An extension of correspondence analysis, CCA is widely used in applied ecology to answer similar questions of species-environment relationships, but has not been previously applied in prehistoric archaeology to explore taxonomic and climatic variables in conjunction. The analyses reveal that the changes in plant and animal exploitation which occurred with the northward dispersal of farmers, crops and livestock correlate with south-north climate gradients, and emphasize the importance of adaptations in the animal domain for the initial establishment of farming beyond the Mediterranean areas.

## Introduction

The distinctness of agricultural economies from such based on foraging is indisputable. Foragers provide their food from plants and animals that are not actively managed, whereas farmers subsist on species which are shaped by a human-driven ecology and reliant on human support and protection. These *domestic* species are so closely incorporated into the cultural sphere that their status as a part of the natural world and their dependence on factors beyond human control are often underestimated. However, duration of sunlight, temperature, rainfall and humidity affect wild and domestic species alike, both directly and, for animals, through the quantity, quality and digestibility of forage. Like their free-living counterparts, domesticates are adapted to specific bioclimatic niches. Problems and failures accompanying the introduction of crops and livestock into new biogeographic zones in the recent centuries, for example in the Americas and Australasia, illustrate this point very clearly [1–5], and domesticate expansions in prehistory must have been similarly challenging.

European agriculture stems from a farming system that emerged in the semi-arid sub-Mediterranean bioclimatic zone of southwest Asia. The initial expansion of domestic species (cattle, sheep, goat, pig, wheat, barley, and a number of leguminous crops) out of this core area was directed longitudinally. Latitudinal dispersal was a later phenomenon, which began approximately two thousand years after domestication and brought farming into environments that increasingly diverged from the areas of domestication as regards precipitation regimes, temperature, seasonality and day length [6]. According to current archaeological evidence, the pioneer settlers who penetrated into the interior of the Balkans by the early sixth millennium calBC were the first to face the challenge of introducing domesticates beyond their native bioclimatic range.

Pioneer settlers can employ a variety of different strategies to cope with unfamiliar ecological conditions, the most proximate being probably a change in favor of crop and livestock species that reproduce best in the new environment. Indeed, the faunal and botanical assemblages from early sixth millennium BC sites in southeast Europe have been shown to vary in the proportions of food plant and animal taxa [7–15]. The impact of environment on these differences in species composition remains poorly explored. This study addresses the relationships between the bioarchaeological assemblages from the first farming sites and their climatic settings. Using multivariate statistical methods, we integrate bioclimatic variables with an extensive dataset of plant and animal remains from Southeast Europe. The resulting comprehension of the species-environment relationships in this key region allows us to infer how cultivation and herding became viable far beyond their original geographic range, and farming subsequently dispersed across prehistoric Europe.

### Environmental and cultural background

**Fig 1:**
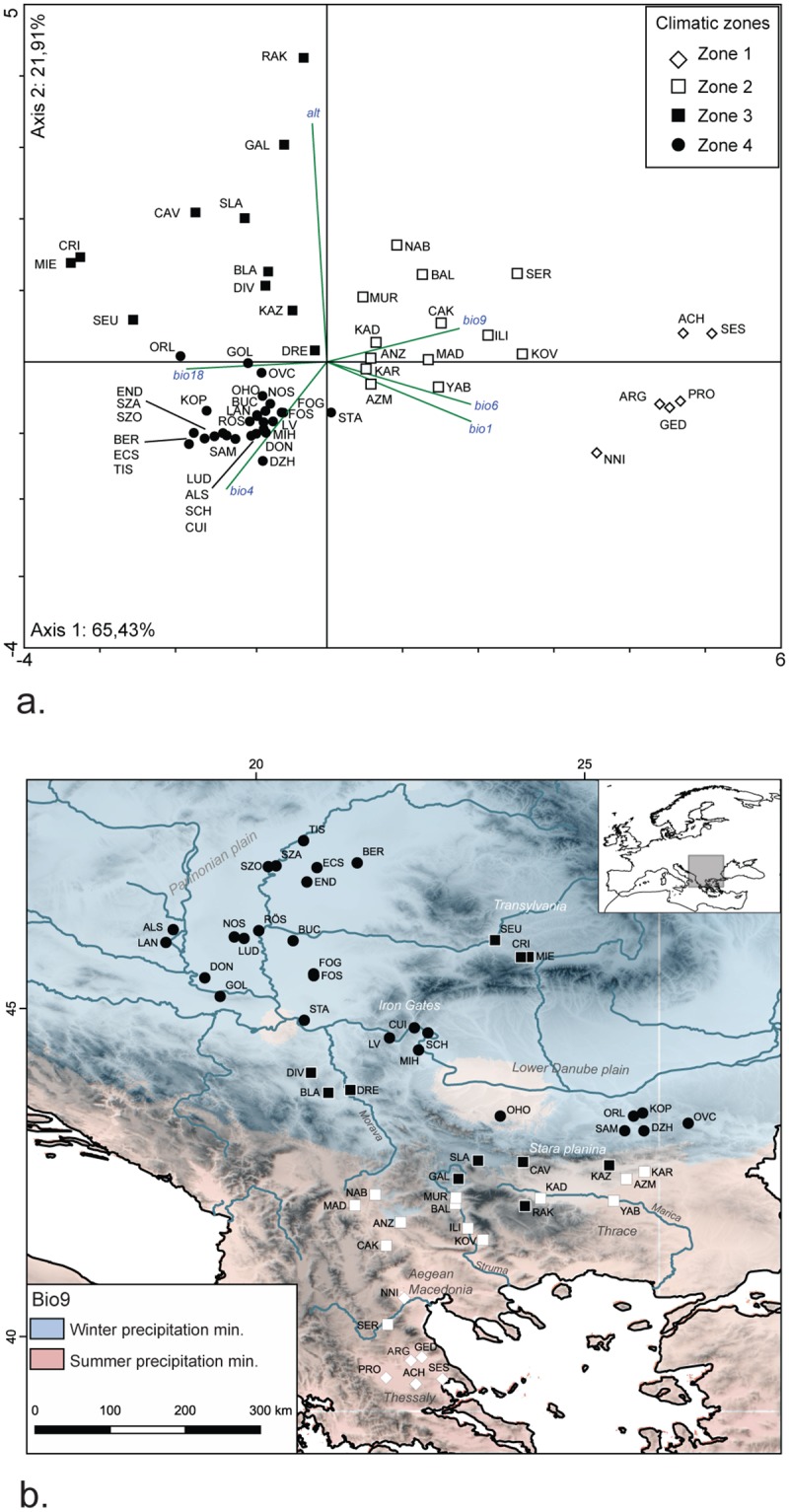
Archaeological sites and their environmental settings. (a) PCA biplot showing grouping of archaeological sites in four clusters (“Climatic zones 1–4”) in relation to altitude and bioclimatic parameters of temperature and precipitation (performed with PAST 3.14). (b) Map showing location of archaeological sites; symbols correspond to the sites’ location in the PCA ordination (“Climatic zones 1–4”). Background: Bio9, data from WorldClim, Global climate data gridded datasource, http://worldclim.org; the map was created using QGIS Version 2.8.9-Wien.

Climate and vegetation in the Balkans vary with latitude and distance from the sea. The Mediterranean ecosystems in the Aegean and Adriatic coastal zones and in the adjacent river valleys of Vardar, Struma, Mesta, and Maritsa, transform through a series of transitional ecotones into temperate continental ecosystems in the vast lowlands of the Pannonian Basin and the Lower Danube. Latitudinal change is reinforced by the mountainous massifs in the interior of the peninsula, which effectively isolate most inland areas from the Mediterranean influence. A major phytoclimatic barrier, dividing the Balkans latitudinally into a southern and northern part, is formed by the Stara Planina Mountains. The areas north of the mountain range feature higher precipitation, longer snow cover, later onset of spring and a diminishing number of thermophilous sub-Mediterranean plant species in comparison to the southern areas [16]. Moreover, the Stara Planina range coincides with the divide in precipitation regimes running across the Balkan Peninsula, with sub-Mediterranean regime characterized by summer dry season (precipitation minimum in August or September) to the south and continental regime with winter dry season (one pronounced precipitation minimum in January) to the north of this border (Fig 1a).

Since the south-north environmental gradient within southeast Europe is dictated by distance from the coast, mountain barriers and change in latitude, a similar gradient likely existed throughout prehistory, even if the exact temperature and precipitation values fluctuated. Pollen-based climate reconstructions for southeast Europe indicate that a gradual rise in summer temperatures up to modern values began around 6000 calBC, while winter temperatures remained close to modern values throughout the Holocene [17]. The only marked shift in temperature and humidity for the period of interest is related to a climate anomaly culminating around 6175–6025 calBC [18, 19], a few centuries after the establishment of the first farming settlements in the coastal plains of Thessaly and Aegean Macedonia [20–23]. This was a period of abnormal seasonal contrasts, with pronounced cooling during winter and a reversing of the seasonal pattern in the Aegean to summers with excessive rainfall and storms [24, 25]. By 6000 calBC, however, climate in southeast Europe had recovered its stability.

Current archaeological evidence pinpoints the plains of Thessaly and Aegean Macedonia as the source of subsequent northward expansion of cultivation and herding into the interior of the peninsula at the turn of the seventh and sixth millennia calBC [21, 26]. Radiocarbon dates indicate a rapid northward expansion starting around 6100/6000 calBC [27]. The archaeological sites from this period have been attributed to a series of cultural groups, defined basically by pottery shapes and decorations (Table 1). The first farming sites in the southern interior of the peninsula (present-day southern Bulgaria and Republic of Macedonia) introduced a bundle of practices and techniques, including long-lived villages with durable above-ground houses and thick occupation deposits with large numbers of pottery sherds, animal bones and carbonized seeds [28–31]. Further northward dispersal (into modern northern Bulgaria, Serbia, Romania and Hungary) was accompanied by a decline in the permanence of site occupation and durability of structures, as well as in the amount and quality of artifacts [32]. These changes have been alternatively attributed to the incorporation of indigenous foraging groups or to adjustments in the pioneer farmers’ lifestyle to environments beyond the Mediterranean climatic influence [7, 33]. The former explanation appears very unlikely in light of recent DNA evidence, which shows a migration from the Aegean with only insignificant genetic admixture of migrants and indigenous hunter-gatherers in the initial phase of farming dispersal across the Balkans and the Pannonian Basin [34–36].

**Table 1:** The earliest farming cultural groups in southeast Europe and their absolute dating.

Geographic region	Archaeological groups/periods	Dating calBC
Thessaly	Aceramic, Early Ceramic, Proto-Sesklo	6500–6000
Aegean Macedonia	Early Neolithic	6500–6000
Vardar valley	Anzabegovo-Vršnik I	6000–5800
	Anzabegovo-Vršnik II-III	5800–5500
Struma and Mesta valleys	Early Neolithic I	6100/6000-5700
	Early Neolithic II	5700–5500
Northern Thrace, Sofia and Pirdop basins	Karanovo I	6000–5700
	Karanovo II	5700–5500
Northeast Bulgaria	Group Koprivets	6000–5700
	Group Samovodene	5700–5500
Morava valley, Vojvodina, Iron Gates	Proto-Starčevo	6100/6000-5800
	Starčevo	5800–5500
Sava and Drava valleys, Transdanubia	Starčevo	5800–5500
Banat, Tisza and Körös valleys	Körös	5800–5500
Lower Danube, Transylvania	Criș I	6000–5700
	Criș II-III	5700–5500

## Materials and methods

The majority of the plant and animal remains found at archaeological sites represent leftovers of food preparation and consumption. Thus, faunal and botanical assemblages reflect the proportions of exploited species, biased to various degrees as a consequence of differential preservation, methods of sampling, identification and quantification. Preservation of bioarchaeological remains depends on their taphonomic history, depositional context and on the surrounding sediments. For example, assemblages from the thick deposits of settlement mounds may show better preservation (leading to higher taxonomic diversity) than those recovered at thin-layered “flat” sites. Cereals are more likely to get preserved in a carbonized state and thus identified in comparison to other plant foods, such as wild fruits (whose seeds are less frequently handled in the proximity of fire), legumes (whose seeds are larger and thus more fragile when charred), or seeds that contain oil. The use of divergent sampling strategies and recovery methods, ranging from judgemental and random to systematic sampling and from hand-recovery to dry and wet sieving with different mesh sizes, as well as different quantification units may lead to divergent results [37–39]. We address these issues of bias and compatibility of raw data from published bioarchaeological reports by selecting sites, samples and species according to a series of criteria, including reliability of dating, secure depositional context, sample size and recovery method (Table A and D in S1 File).

### Site selection

For selection we assessed sites dating to the phase of farming introduction in each geographic region. Designated as “Early Neolithic” in the archaeological terminology, this phase shows an offset in terms of absolute dates owing to the south-north delay in the inception of farming (c. 6500–6000 calBC in the Aegean, c. 6100-5700/5500 calBC in the remaining regions). Radiocarbon dates are available for most sites, whereas the remaining have been assigned to the period of interest by relative archaeological dating. Criterion for inclusion of sites in the dataset was the availability of primary data, comprising published reports with seed counts for the plants and with number of identified specimens (NISP) for the fauna.

### Archaeobotanical materials

The seed counts were taken from the original publications. To ensure comparability, only charred seeds, not chaff and imprints in clay, were included in the dataset. All assemblages were recovered by flotation, with the exception of Nea Nikomedia, Gediki and Prodromos 1–2 (comprising hand-collected visible plant remains). The latter were nevertheless included to enlarge the small sample from the Aegean.

From the range of remains present at a site, the most common edible plants were selected. These include the domestic crops and the most frequent gathered nuts and fruits (Table 2). Although a range of seeds listed as “potential weeds” or “others” in the archaeobotanical reports could also qualify as edible, these were not considered in the present study because data on such taxa were available only for a small subset of the sites. Identifications were taken from the original publication, with the following few exceptions due to differences in employed taxonomic categories. (1) Because of the problematic distinction between free-threshing tetraploid and hexaploid wheat grains, seed numbers of all free-threshing wheats were combined in the category „*Triticum aestivum/durum*“. (2) Doubts in the reliability of identification of spelt grains caused the elimination of this cereal plant. (3) Identifications of different barley, lentil and field pea varieties were summarized to genus level, since species were distinguished only for a minority of the assemblages. Rye (*Secale cereale*) and oats (*Avena sativa*) were not considered because of their very likely status as weeds. Millet (*Panicum miliaceum*), present as single seeds in several samples, was excluded from the dataset because the cultivation of this crop in Europe before c. 1500 calBC is still insufficiently proven [40].

**Table 2:** List of edible plant taxa included in the archaeobotanical dataset.

Taxon/Group	Original identification	Common name
**Cultivated food plants**		
*Triticum monococcum*	*Triticum monococcum*	Einkorn
*Triticum dicoccum*	*Triticum dicoccum*	Emmer
*Triticum aestivum/durum*	*Triticum aestivum*, *Triticum aestivum compactum*, *Triticum durum*	Free-threshing wheat
*Hordeum vulgare*	*Hordeum* sp., *Hordeum vulgare* var. *vulgare*, *Hordeum vulgare* var. *nudum*	Hulled and free-threshing barley
*Cicer arietinum*	*Cicer arietinum*	Chick pea
*Lathyrus sativus*	*Lathyrus sativus*, *Lathyrus sativus/cicera*	Grass pea
*Lens*	*Lens* sp., *Lens culinaris*, *Lens esculenta*	Lentils
*Linum*	*Linum* sp., *Linum usitatissimum*	Flax
*Pisum*	*Pisum* sp., *Pisum sativum*	Field pea
*Vicia ervilia*	*Vicia* sp., *Vicia ervilia*	Bitter vetch
**Wild edible plants**		
*Amygdalus communis*	*Amygdalus communis*	Almond
*Ficus carica*	*Ficus carica*	Fig
*Pistacia*	*Pistacia* sp., *Pistacia terebinthus*, *Pistacia atlantica*, *Pistacia vera*	Pistachio
*Cornus mas*	*Cornus mas*	Cornelian cherry
*Cornus sanguinea*	*Cornus sanguinea*	Common dogwood
*Corylus avellana*	*Corylus avellana*	Hazelnut
*Fragaria*	*Fragaria* sp., *Fragaria vesca*	Strawberry
*Malus / Pyrus*	*Malus* sp., *Malus pumila*, *Malus sylvestris*, *Pyrus* sp.	Crab-apple, pear
*Physalis alkekengi*	*Physalis alkekengi*	Bladder cherry
*Prunus*	*Prunus* sp., *Prunus institia*	Plum, bullace
*Prunus mahaleb*	*Prunus mahaleb*	St. Lucie cherry
*Prunus spinosa*	*Prunus spinosa*	Blackthorn
*Quercus*	*Quercus* sp., *Quercus robur*	Acorn
*Rubus*	*Rubus* sp., *Rubus idaeus*, *Rubus fruticosus*, *Rubus caesius*	Raspberry, blackberry
*Sambucus*	*Sambucus* sp., *Sambucus nigra*, *Sambucus ebulus*	Elder, dwarf elder
*Trapa natans*	*Trapa natans*	Water chestnut
*Vitis*	*Vitis* sp., *Vitis vinifera* subsp. *sylvestris*	Grape

Samples determined in the original report as storage finds were omitted from the total seed counts because the size of such depositions can skew the seed counts towards particular taxa. For each site, assemblages from different years and contexts but from the same phase or from sub-phases were merged; if different investigators were involved, the largest sample was taken (to ensure consistency of identification methods between the individual assemblages). After excluding the elements specified above and adding the seed numbers to phases, sites with fewer than 50 items were omitted to ensure representativeness.

The final botanical dataset with abundance data encompassed 29 sites with 51,799 items (Table B in S1 File). Presence-absence values were determined for each of these 29 sites. Additionally, another 12 sites with only presence-absence data were considered when assessing plant taxonomic diversity across the ecological zones (Table C in S1 File).

### Zooarchaeological materials

The faunal dataset contains assemblages from published reports including NISP (number of identified specimens). Four of the faunal assemblages were obtained by sieving, ten were hand-collected and for 27 assemblages the collecting method was not specified in the publication (Table D in S1 File). Because sieving has been an exception rather than a rule of archaeological practice in the Balkans, especially in the past, the collection of faunal remains by sieving is normally indicated in the publication. Therefore, the majority of the assemblages with unspecified collection method (most of which are derived from older excavations) were presumably also hand-collected.

In the present study we use NISP rather than other quantitative units such as MNI (minimum number of individuals) or MNE (minimum number of [skeletal] elements). While NISP are primary data, MNI and MNE represent derived data that are partially based on the NISP. As researchers may differ in their approaches to calculate MNI / MNE, the obtained values are not always comparable [41]. Moreover, it has been shown that MNI values can be accurately predicted by the NISP, and that MNI or similar quantitative units are not superior to NISP in providing estimates of taxonomic abundances [42]. Nevertheless, it should be kept in mind that both units provide only an *estimate*, and not an actual *measurement*, of the assemblage’s taxonomic composition.

From the range of identified species, only the large and middle-sized meat-bearing mammals, identified to species level, were included in the dataset. Exception is the category „sheep/goat“, which summarizes specimens identified to genus and the unidentifiable remains of either sheep or goat (Table 3). Specimens with unknown wild or domestic status (e.g. *Bos* sp., *Sus* sp.) have not been included in the dataset. In case antler fragments were reported separately, their numbers were not included, as they might have been collected and brought to the sites as a raw material. Equids were omitted from the dataset either, since their remains are absent or very rare at all but two sites (Nosa and Röszke). The latter represented significant outliers that exerted a disproportionate influence on the results. This reduced range of species is suitable for investigating the proportions of primary meat sources in diet, which is the focus of our study. Variety of meat sources, and in particular differences in the exploitation of small (e.g. lagomorphs, birds, fish, mollusks) as opposed to large taxa, was not considered, as the presence of small animals in the faunal assemblages was largely dependent on the practices of recovery and was not of comparable quality for all sites.

**Table 3:** List of main meat animals included in the zooarchaeological dataset.

Taxon/Group	Original identification	Common name
**Livestock**		
Ovis/Capra	Caprinae, Ovicaprinae, *Capra/Ovis*, *Ovis aries*, *Capra hircus*	Sheep/goat
*Bos taurus*	*Bos taurus*, *Bos primigenius* f. taurus	Cattle
*Sus domesticus*	*Sus domesticus*, *Sus scrofa* dom.	Pig
**Large-/middle-sized game**		
*Cervus elaphus*	*Cervus elaphus*	Red deer
*Capreolus capreolus*	*Capreolus capreolus*	Roe deer
*Bos primigenius*	*Bos primigenius*	Aurochs
*Sus scrofa*	*Sus scrofa*	Wild boar

Bone counts were collapsed on the site-phase level. Assemblages were merged when coming from different excavation years and contexts within the same chronological phase (or sub-phases) and studied by the same investigator. If data was published by different investigators, the largest sample was taken to ensure consistency of identification methods within the sites. After combining sub-phases and omitting the taxa specified above, only sample sizes >500 NISP were retained to maximize representativeness. Data selection resulted in 41 faunal site samples with 115,142 items (Table D in S1 File).

The final quantitative dataset comprised 57 sites, for 29 of which there were seed counts and for 41 bone counts.

### Bioclimatic data

Because the present-day south-north environmental gradient in southeast Europe is determined by distance from the coast, mountain barriers and latitude, we cautiously assume that it existed throughout the past (even when the exact values of temperature and precipitation fluctuated). As we did not wish to identify absolute climatic predictors, but were only interested in the relative differences along this climatic gradient, we chose to use high-resolution modern climate data. Spatially interpolated climate data were obtained from the WorldClim—Global climate data gridded datasource (http://worldclim.org) at a spatial resolution of 30 arc s (1-km) [43]. The data are based on records from 1950–2000. Values for altitude and five bioclimatic parameters were extracted for each site with a botanical or faunal assemblage, based on its geographic coordinates and using QGIS 2.8.9-Wien (Table F in S1 File). The five bioclimatic parameters are derived from the monthly temperature and rainfall values; they highlight annual trends in temperature and precipitation, as well as extreme or limiting environmental factors relevant to cultivation and herding (Table 4). Derived bioclimatic parameters are considered to correspond more directly with species physiology in comparison to monthly values, and are widely employed by biologists and ecologists in species distribution modeling [44].

**Table 4:** Biogeographic variables and description of their relevance for cultivation and herding.

Code	Name	Description
Bio1	Annual Mean Temperature	In °Celsius, a measure of the total energy inputs for the ecosystem
Bio4	Temperature Seasonality	Standard deviation of monthly mean t*100, a measure of temperature change over the course of the year
Bio6	Minimal Temperature of Coldest Month	In °Celsius, indicates cold temperature anomalies throughout the year which can affect plant and animal growth (cold stress)
Bio9	Mean Temperature of Driest Quarter	In °Celsius, provides mean temperatures during the driest three months of the year (indicating summer vs. winter rain maximum); related to seasonality of plant growth cycles
Bio18	Precipitation of Warmest Quarter	In millimeters, provides total precipitation during the warmest three months of the year; related to seasonality of plant growth cycles (drought stress)
Alt	Altitude	In meter a.s.l.

### Data analysis

The large and heterogeneous dataset with altitude, five bioclimatic and 30 bioarchaeological variables for 57 archaeological sites was analyzed using ordination techniques. Ordination is a standard technique in modern applied ecology, used for exploring complex biological assemblages of species to elucidate issues similar to these of our study: grouping of assemblages in relation to environment, geographic gradients, and associations between species communities and ecology.

1. To discriminate between sites with similar ecology, principal-component analysis (PCA) was applied to the bioclimatic parameters extracted for each of the 57 archaeological sites. PCA is one of the most widely used variable-reduction techniques, summarizing an initial large set of variables to a smaller set of derived (“synthetic”) variables called “principal components” or “eigenvectors”.
2. To explore the relationship between climate and plant/animal abundance data (seed numbers and bone NISP), we applied Canonical Correspondence Analysis (CCA) with climate as explanatory variable and species and sites as response variables. CCA is an extension of correspondence analysis widely employed in applied ecology to explore the relationship of biological assemblages with their environment [45]. To separate the species according to their ecological niches, an extra restriction that the ordination axes are linear combinations of the environmental variables is imposed in CCA. The resultant ordination diagram consists of vectors for environmental variables and points for species and sites. The ordination visualizes the „realized niches”of different species: in purely ecological datasets, these niches are shaped by species competition, whereas for bioarchaeological assemblages, human decisions play the main role. Data arrangement was interpreted in this study according to the “distance rule” (sites plotting close to each other are similar in their data structure) and the “centroid principle” (sites plotting close to a species point have higher abundance than those far from the species point) [45].
3. In addition, Non-Metric Multidimensional Scaling (NMDS) analysis was carried out to bring out patterns in the crop assemblages, which failed to show structuring in CCA. NMDS is not an eigenvector technique like PCA or CCA, which arrange the data along synthetic axes of variance. Instead, in the NMDS ordination plot samples are placed in a two-dimensional coordinate system so that similar samples are close and the original ranked differences between the samples are preserved.

Prior to analysis, the rare plant taxa (present in less than 10% of the site samples) were summarized to minimize noise: *Pistacia*, *Ficus carica* and *Amygdalus communis* were combined into a group of „Mediterranean fruits“; all *Prunus* species were combined in the group „Prunus“. The raw counts of seeds and bones per taxon were transformed logarithmically to prevent ordinations that reflect primarily sample size; to avoid taking the logarithm of zero, 1 was added to each raw count. PCA, CCA and MNDS analyses were performed with the PAST 3.14 software [46].

## Results

### Bioclimatic zonation of sites

Principle-component analysis (PCA) of altitude and five bioclimatic variables (Bio1, Bio4, Bio6, Bio9 and Bio18, Table 4) was carried out to discriminate clusters of sites with similar climatic settings. A projection of the original variables onto the ordination diagram shows that Axis 1 is determined by the five bioclimatic variables, being positively correlated with Bio1, 6 and 9 and negatively with Bio4 and 18, while the second eigenvalue (Axis 2) correlates positively with altitude (Fig 1a). The biplot indicates a differentiation between warmer climate with mild winters and dry summers and cooler climate with stronger seasonality of temperature and moist summers. The sites separate in four main clusters in relation to these climatic trends. These four clusters (“zones”) were used as classes in further analysis of the botanical and faunal data (Table 5).

**Table 5:** Grouping of archaeological sites in four clusters (“zones”) in PCA in relation to altitude and bioclimatic parameters.

Zone	Conditions	Location of sites
1	Mediterranean conditions with hot dry summers and mild rainy winters	Littoral areas of Thessaly and Macedonia
2	Transitional sub-Mediterranean environments with lower annual mean temperature and stronger seasonality	River valleys of Vardar, Aliakmon, Lower Struma and Maritsa
3	Sub-continental environments, cooler temperatures	Higher altitude intra-mountain basins or valleys—the basins of Sofia, Zlatitsa-Pirdop and Kazanluk, the valleys of Mesta, Upper Struma, Middle Morava and its tributaries, and the Transylvanian high plateau
4	Continental climate with cold dry winters and moist early summers	Lowlands of the Pannonian plain and the Lower Danube

### Plant taxa and environment

**Fig 2:**
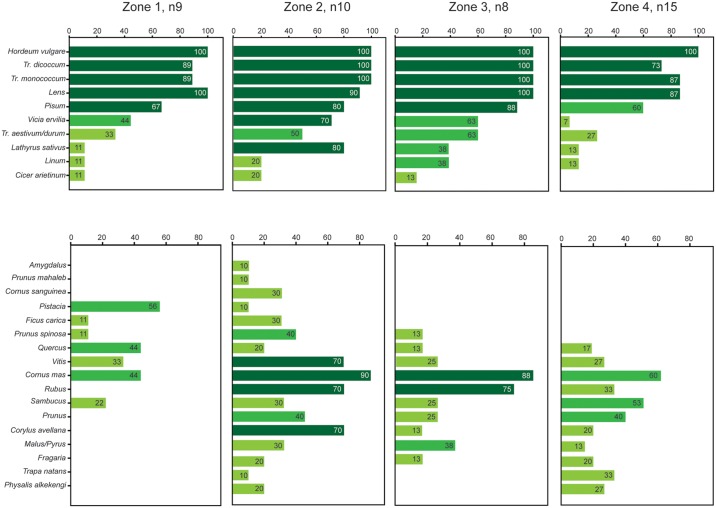
Presence of plant taxa at sites. Numbers in the bars show per cent of sites in each zone at which a taxon was present.

The presence/absence data of domestic and wild taxa indicates differences between the four zones, with wider plant spectra in Zones 2 and 3 (Fig 2). It highlights a group of “basic” crops, comprising barley, emmer, einkorn, lentils and field peas, which were present in the vast majority of samples across all four zones. The remaining crops, i.e. free-threshing wheat, flax, grass pea, chick pea and bitter vetch, were found in a lower proportion of the samples. The spectrum of wild taxa reflects adaptations of foraging for plant foods to more northern ecosystems. Spatially restricted thermophilous taxa, such as pistachio (*Pistacia*) and fig (*Ficus carica*), were gradually replaced by cornelian cherry (*Cornus mas*), hazelnut (*Corylus avellana*) and berries (*Sambucus*, *Rubus*) as the most widely exploited wild foods of the interior, while water chestnut (*Trapa natans*) grew in importance in the northernmost areas. In terms of both cultivated and wild food plants, Zone 2 stands out with a remarkably high taxonomic variety at most sites.

**Fig 3:**
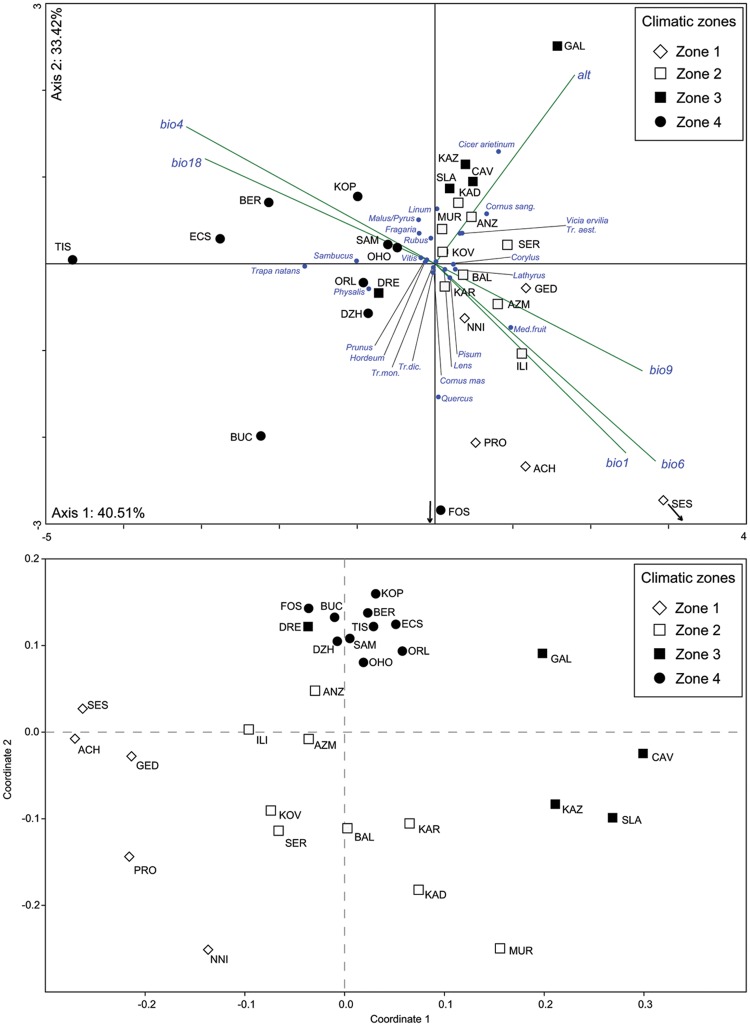
Grouping of archaeobotanical samples in relation to climate. (a) CCA ordination based on seed counts of all wild and domestic taxa. (b) NMDS ordination based on seed counts of domestic taxa only (CCA and NMDS performed with PAST 3.14).

Canonical Correspondence Analysis (CCA) was carried out on a dataset with altitude, five bioclimatic variables, 29 site-samples (log transformed seed counts) and 23 plant taxa/groups. The first eigenvector (Axis 1) is a climatic gradient determined by the five bioclimatic variables and accounts for 40.51% of the total variation in plant composition, while the second is determined by altitude. In the resultant ordination (Fig 3a), most crops are located near the point of origin. Among the cereal crops, barley (*Hordeum* sp.) plots relative to Bio 4 and bio 18 (cooler climate with moist summers) and free-threshing wheat (*Tr*. *aestivum/durum*) tends towards warmer climate and higher altitude. All leguminous crops show positive values on Axis 1 and association with Bio1, 6 and 9 (summer drought, mild winters). Several wild taxa are distinguished on Axis 1 in correlation with the bioclimatic variables. Species such as common dogwood (*Cornus sanguinea*) and “Mediterranean fruits” exhibit positive values and an association with Bio1, 6 and 9 (“Mediterranean” regime with summer drought, mild winters), while water chestnut (*Trapa natans*), bladder cherry (*Physalis alkekengi*), elder (*Sambucus*), apple/pear (*Malus/Pyrus*) and berries (*Fragaria*, *Rubus*) have negative values and correlate with Bio4 and Bio18 (“continental” regime with pronounced temperature seasonality, summer precipitation maximum).

The points for sites of Zones 1, 2 and 3 are positively associated with Axis 1 and correlate with “Mediterranean” regime and plant taxa. In contrast, sites of Zone 4 are separated by their exclusively negative values, which position them relative to the “Continental” regime and plant taxa. When wild taxa are excluded from the CCA, however, this structuring of the dataset along a climatic gradient is obscured, indicating that climate is not the sole factor accounting for the variance in crops (Plot B in S2 File). A non-metric multidimensional scaling (NMDS) analysis was carried out with crop taxa and bioclimatic variables to address this issue (Fig 3b). Contrary to CCA, NMDS analysis does not impose the restriction that climate variables determine the ordination and clustering is thus dictated by a combination of bioclimatic variables and abundances. In the resulting plot samples from Zones 1, 2 and 3 are arranged in discrete groups of loosely scattered sample points. In contrast, the samples of Zone 4 form a clearly distinguishable, tight cluster which includes sites from very distant geographic locations and with distinct cultural affiliations (Körös, Starčevo and Criș groups), but with similar climatic background.

### Animal taxa and environment

**Fig 4:**
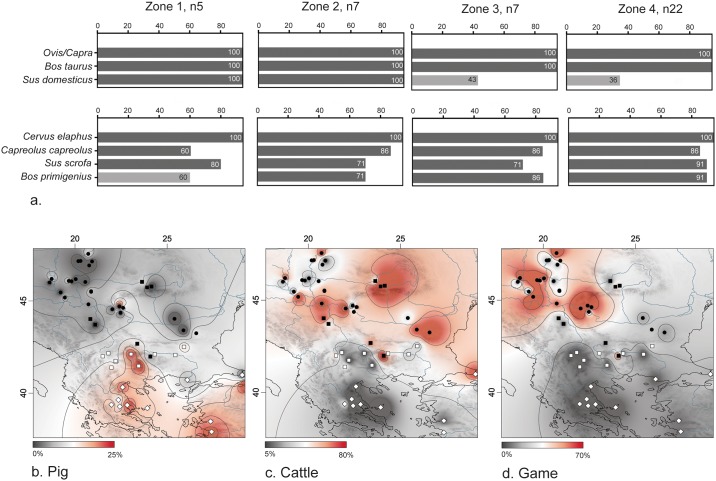
Presence and geographic distribution of wild and domestic animal species. (a) Presence of animal taxa at sites. Numbers in the bars show per cent of sites in each zone at which a taxon was present (with a threshold of 5% from domestic / wild NISP respectively). (b) Pig frequencies at sites (expressed as per cent from NISP of domestic animals). (c) Cattle frequencies at sites (expressed as per cent from NISP of domestic animals). (d) Frequencies of large and medium-sized wild mammals (expressed as per cent from NISP). The maps (b)-(d) include eight sites which are not part of the main dataset because of incomplete data or location outside the area of interest (listed in Table E in S1 File). Frequency data were mapped and interpolated by Inverse Distance Weighting (IDW) using QGIS Version 2.8.9-Wien.

Presence/absence data reveals little inter-regional variation in the range of exploited animal taxa, with the notable exception of domestic pigs, whose abundance drops abruptly in Zones 3 and 4 (Fig 4a). To bring out spatial patterning in taxonomic compositions, ratios of sheep/goat, cattle and pigs (as per cent of the NISP of these three species) and of wild taxa (as per cent of the total NISP) were calculated from the primary faunal dataset and interpolated by Inverse Distance Weighting (IDW) to create a continuous surface and derived contours from the discrete site points (Fig 4b–4d). The maps reveal clear south-north differences, pig herding being popular only in Mediterranean environments, cattle and large game gaining in importance toward north and northwest respectively. Although the very high proportions of cattle at a few sites in Transylvania may exaggerate their importance in this region, the interpolations are in line with the trend of increasing cattle exploitation beyond the Aegean littoral and Thrace noted by other authors [8].

**Fig 5:**
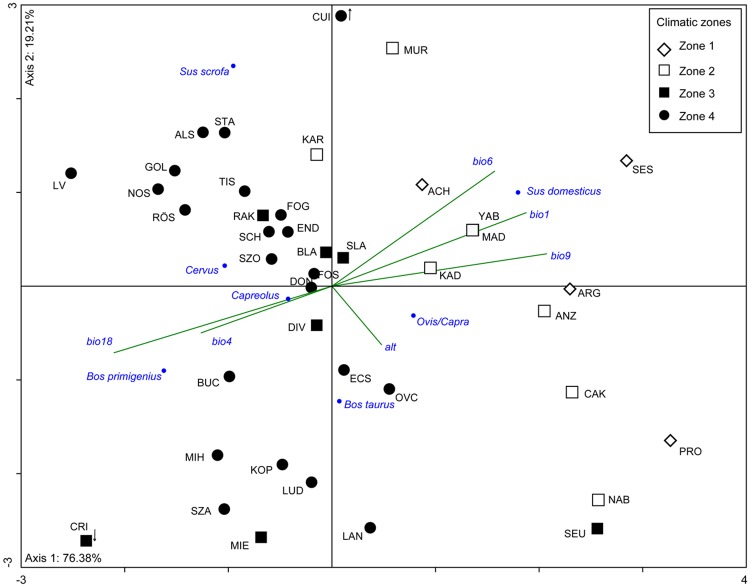
CCA ordination based on bone counts of wild and domestic taxa. CCA performed with PAST 3.14.

The CCA, using altitude, five bioclimatic variables and abundance data for 41 sites and 7 animal taxa, confirms that these spatial trends are climate-related. The first synthetic gradient (Axis 1) is determined by the five bioclimatic variables and arises from a contrast between “Mediterranean” regime with summer drought and mild winters (increasing values of Bio1, 6 and 9) and “continental” regime with pronounced temperature seasonality and a summer precipitation maximum (increasing values of Bio4 and 18). Axis 1 accounts for 76.38% of the total variation in the faunal composition, and differentiates between domestic taxa, having positive values, and wild taxa with negative values. The positioning of the site points in relation to the faunal taxa and environmental variables separates sites of Zones 1 and 2 from those of Zones 3 and 4 (Fig 5). The former have mostly positive values on Axis 1 and are associated with sheep/goats and pigs. The ordination of Zone 3 and 4 sites on Axis 1 shows that cattle and large game were better represented in these samples. Although the very high proportions of sheep at some Zone 4 sites (i.e. Ludas, Ecsegfalva, Lanycsók and Endröd) do not seem to exert significant influence on the ordination, they still deserve particular attention. The causes for the abundant sheep remains at these sites have been a matter of debate [47–49] because the swampy environments of the Danube and Tisza plains are not suitable for sheep husbandry. Problems with livestock overwintering due to snow cover and a shorter plant growing season may account for the surprising prevalence of sheep [50]. In comparison to their southern neighbors, pioneer herders in these northern areas were possibly forced to slaughter more animals before or during winter due to fodder shortages. Where hunting was not a reliable buffer, caprines could have been preferred over cattle because of their faster reproduction and higher annual growth rate. Notably, the “caprine” sites in Zone 4 are situated within open grassland areas, where large game hunting must have been much less rewarding in comparison to the forest margins [51]. Other suggested explanations include cultural conservatism [48] and a slower process of adaptation [14].

## Discussion

Past subsistence systems give rise to abundant and heterogeneous bioarchaeological remains. To uncover trends in such complex datasets and to situate them within their environmental contexts, multiple variables need to be assessed simultaneously. Multivariate ordination techniques, such as correspondence analysis (CA), have been repeatedly employed for quantifying and visualizing distribution patterns within archaeobotanical and zooarchaeological remains [14, 52–57]. Their potential for detecting quantifiable relationships between environmental and bioarchaeological variables, however, has remained underexplored. In this study, we applied canonical correspondence analysis (CCA, an extension of correspondence analysis), using bioclimatic data and a comprehensive bioarchaeological dataset, to revisit the environment-species relationship for the initial phase of farming in southeast Europe. The results demonstrate a climate-related change in agriculture with the northward dispersal of farmers, crops and livestock, highlighting several notable trends:

### Crop diversity fluctuated in relation to climate

The lack of structuring in the CCA ordination of the crop samples indicates that crop taxonomic proportions do not directly correspond to climate. The most likely explanation for this counterintuitive outcome stems from plant physiology. In the semi-arid Mediterranean environments (Zone 1), crops such as the early domestic cereals and pulses could retain the growth habit of their wild progenitors, which germinate in autumn to take advantage of winter moisture, and flower early in spring to complete grain filling before the summer droughts. Mediterranean farmers probably had a range of strains with various degrees of drought resistance to choose from. In the interior of the Balkans (Zones 2–4), however, the critical factor for cultivation shifted gradually from summer droughts to winter / spring frosts. Winterkill (a severe damage of crops due to long-lasting freezing conditions, snow cover and mold infections) as well as late spring frosts can be devastating for winter-sown crops. Frost after initiation of flowering parts is particularly damaging—at this stage, even the hardiest modern races do not withstand temperatures lower than several degrees below 0°C [58, 59]. In the pioneer phase, all early crop strains coming from the Mediterranean were likely ill-adapted to cold-induced infections and late frosts. Simple change of taxonomic composition in favor of particular crops must have been therefore ineffective as a means of avoiding climate-related failures.

In the past as in the present, the most efficient strategy of minimizing frost damage (apart from growing improved varieties) has been the simultaneous cultivation of many crops to ensure a wide span of flowering dates. Indeed, presence data show a very wide spectrum of crops at the majority of the sites in Zones 2 and 3, where both late frosts and summer drought limited the reliability of cultivation (Fig 2a). Diversification was reversed in Zone 4, in which a summer precipitation maximum removed drought as a limiting factor and many of the drought-resistant leguminous crops became rare. Samples of Zone 4 form a very tight group in the NMDS ordination plot (Fig 3b), emphasizing their distinctiveness in relation to the assemblages from other zones. Zone 4 crop spectra in the later sixth millennium calBC remained similar, with somewhat higher visibility of crops such as free-threshing wheat, bitter vetch and flax in the botanical assemblages from the central Balkans [60, 61].

### Climate was an important factor for the farmers’ choice of domestic and wild animals

While the preference for small livestock and pig in Zones 1–2 and the increasing importance of cattle and hunting in Zones 3–4 have been noted in previous research [8, 52, 62], their relationship to climate has not been addressed directly. We could demonstrate that climate accounts for the main part of the taxonomic variation in the faunal assemblages. Sheep and goats do not have wild relatives in the Balkans and likely experienced feeding problems in forested environments with winter snow cover. Moreover, sheep and goats are seasonal breeders having a reproductive cycle determined by photoperiod [63, 64] and, with increasing latitude and later spring onset, they would potentially give birth too early. An early birth season not only threatens newborns’ survival, but might also decrease the productivity of herding: for modern-day sheep breeds, a 1°C decrease in mean spring temperature has been estimated to reduce lamb autumn body mass by nearly 0.4 kg [65]. Mediterranean cattle probably also suffered acclimation difficulties and fall in productivity when first brought into the interior. However, cattle reproduction is not constrained by photoperiod and they benefit from feeding behaviors and physiology similar to their free-living local relative, the aurochs. The high proportions of large game at many Zone 4 sites underline the first farmers’ preference for species that are well adapted to the local environment. In the later sixth and the fifth millennia calBC, cattle becomes predominant among domesticates throughout the northern Balkans and the Pannonian basin, and the contribution of hunting remains high [13, 14].

### Pig husbandry was nearly abandoned in spite of the favorable environment

The decline in pig herding is not readily explained by environment, as pigs should have thrived in the forests of interior southeast Europe. Decrease in pig frequency may be related to traditional practices in herding and feeding pigs. Although data on pig diet from the Balkans are still few and far between, stable isotope values hint that a specific strategy of close control was characteristic for the earliest pig husbandry [66–68]. With the decline of settlement permanence in the northern parts of the area, opportunities for keeping these animals as “house pigs” on garbage in and near the settlements diminished. So far, the practice of free-range forest herding has not been demonstrated for the initial phase of farming. Notably, the abundance of pigs in the faunal assemblages from the central Balkans and parts of the Pannonian basin rose sharply in the last centuries of the sixth millennium BC, and at numerous sites domestic pigs ranked second after cattle among domestic animals [13, 14]. In the plains of the Lower Danube pig herding increased in importance somewhat later, around the middle of the fifth millennium BC [69]. For both regions, this trend coincided with the establishment of larger and more permanent sedentary communities.

### Changes in animal husbandry played a primary role in the dispersal of farming

Because animals can be moved, herding allows more flexible responses in unfamiliar and unfavorable environments in comparison to plant cultivation. Once the fields are sown with the crop varieties at hand, the cultivators’ opportunities to limit crop damage by frost or cold-related pathogens are limited, whereas herders can intervene at many stages of the reproductive cycle to enhance animal productivity. Options include, for example, changing pastures, stalling and foddering of young and lactating individuals, selective culling, or intensified milk exploitation. Thus, in unfavorable conditions decline in the crop sector of food provision can be offset by investment in the more resilient animal sectors, including both herding and hunting. Reliance on animals is well documented for various historical contexts as a strategy of survival during dispersal into unfamiliar environments [4].

**Fig 6:**
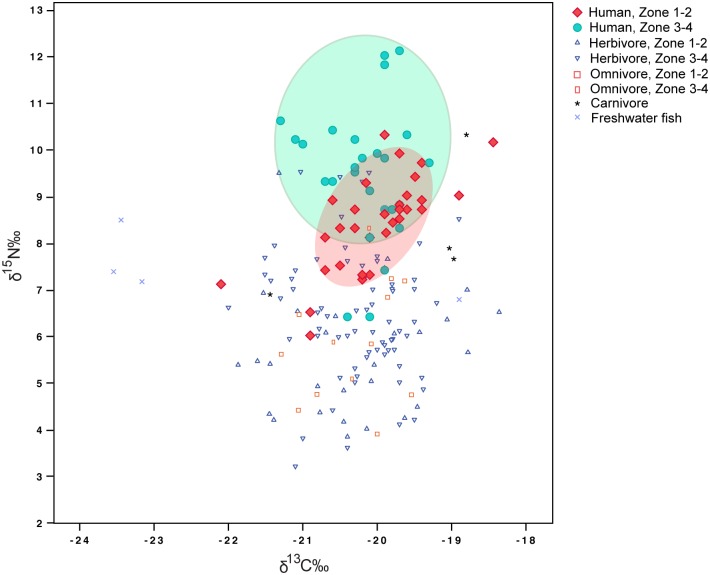
Carbon and nitrogen stable isotope values of humans and animals from southeast Europe. Humans from Zones 3–4 are significantly enriched in δ^15^N relative to those from Zones 1–2 (*t*-test, p = 0.01); the δ^13^C are not significantly different. For data see Table G in S1 File.

Our results emphasize that animal exploitation followed more closely the climatic gradient of the Balkans compared to crop husbandry. Evidence from other dietary proxies, such as organic residues in ceramic vessels and stable isotope values of human skeletal remains, also corroborates an increasing reliance on animals with dispersal of farmers beyond the zone of Mediterranean influence. A recent study of organic residues in pottery has demonstrated a marked divergence between the (sub)Mediterranean and temperate regions of Southeast Europe, revealing a particularly strong dairying signal in the biochemical record from sites in present-day central Serbia and Hungary [50]. In general, the available lipid evidence suggests that milk was exploited to some degree by the first farmers in our Zones 1 and 2 [50, 70] but milking markedly intensified at sites in Zones 3 and 4 [50]. We plotted the existing stable isotope dietary data from southeast Europe to examine whether the northward dispersal of farming coincided with increasing proportions of protein in human diets (Fig 6, for data sources see Table G in S1 File). Humans from Zones 3–4 show enriched δ^15^N values (two-sample *t*-test, p = 0.01), the most probable explanation for which are higher consumption of animal proteins with a considerable aquatic component for some of the analyzed individuals. Another potential cause for the elevated δ^15^N are higher levels of crop manuring in Zone 3 and 4, a factor which is also related directly to the animal sector of the economy [71, 72]. Although evidence for the degree of integration of crop and livestock husbandry among the pioneer farmers in the Balkans is very scarce [72], research in other regions has demonstrated that collection and spread of manure and the use of cattle for draught and transportation were part of the earliest farming systems in Europe [68, 72–75].

## Conclusions

The growth and reproduction cycles of domestic plants and animals are attuned to meet human demands within anthropogenic environmental niches. Yet, even when crops and livestock are intimately enmeshed in the cultural sphere, they remain dependent on factors beyond human control, such as duration of sunlight, temperature, and rainfall. It has been noted in previous research that the earliest Old World domesticates were embedded in a Mediterranean ecosystem and that their dispersal to higher latitudes in the interior of Europe likely entailed adjustments in husbandry practices as well as genetic adaptations [76–80]. The early sixth millennium calBC pioneer settlers in the interior of the Balkans were among the first to face these challenges. With its gradual latitudinal transition from Mediterranean to continental conditions, the Balkans are a key region for understanding the expansion of plant and animal husbandry into the interior of Europe.

Pioneer farmers can respond in various ways to unfamiliar ecological conditions, most easily by adjusting the species mix in favor of crop and livestock taxa that reproduce best in the new environment. This study explored the relationship between climate and the taxonomic frequencies of domestic and wild food species in bioarchaeological assemblages from the first farming sites in southeast Europe. A dataset including altitude, five bioclimatic and 30 bioarchaeological variables (plant and animal taxa) for 57 archaeological sites was analyzed using ordination techniques. By revealing complementary adjustments in the plant and animal sectors of the farming system, our results emphasize the importance of assessing the botanical and faunal datasets in conjunction. The analysis of crop assemblages did not show clear associations between particular crop taxa and bioclimatic zones. When confronted with the sub-Mediterranean ecosystems of the southern Balkans (in modern-day southern Bulgaria and Republic of Macedonia), the pioneer farmers apparently chose a strategy of diversification. Most communities in this zone exploited a very broad spectrum of crops, possibly as a means of reducing climate-related losses. Further latitudinal expansion into areas with increasingly continental conditions in the northern Balkans and the Pannonian Basin (modern northern Bulgaria, Serbia, Romania and Hungary), however, resulted in the abandonment of many leguminous crops, and the spectrum of cultivated plants at most sites was reduced. In contrast to the crop dataset, the taxonomic proportions in the faunal assemblages are clearly structured by climate: the frequency of sheep, goats and pigs diminishes, while cattle and wild species gain in importance with the northward expansion of farming. In conjunction with other lines of dietary evidence, such as organic residues in ceramics and stable isotope values in human bones, these results suggest that the animal domain was of key importance for the initial establishment of farming beyond the Mediterranean areas.

## Supporting information

S1 File The file contains Tables A-H. Table A-C, Archaeobotanical dataset. Table D-E, Zooarchaeological dataset. Table F, Site coordinates and bioclimatic data. Table G, Stable isotope values (δ^15^N and δ^13^C) of humans and animals. Table H, References.
(XLSX)

S2 File **The file contains Table A** Scores for the first six factors for Fig 1. **Table B**, Scores for the first six factors for Fig 3a. **Table C**, Scores for the first six factors for Fig 5. **Table D.** Scores for Fig 3b NMDS ordination based on seed counts of domestic taxa only. **Plot A.** Shepard plot for Fig 3b, NMDS ordination based on seed counts of domestic taxa only. **Plot B**, CCA ordination based on seed counts of domestic taxa.
(PDF)
