# Correction: Pioneer farming in southeast Europe during the early sixth millennium BC: Climate-related adaptations in the exploitation of plants and animals

**DOI:** 10.1371/journal.pone.0202668

**Published:** 2018-08-15

**Authors:** Maria Ivanova, Bea De Cupere, Jonathan Ethier, Elena Marinova

Two references are omitted from the from the second sentence of the third paragraph in the Introduction section.

The sentence should read: Indeed, the faunal and botanical assemblages from early sixth millennium BC sites in southeast Europe have been shown to vary in the proportions of food plant and animal taxa [7–15] (Kreuz et al., 2005) (Kreuz et al., 2017).

The references are:

Kreuz A, Marinova E, Schäfer E, Wiethold J. A comparison of early Neolithic crop and weed assemblages from the Linearbandkeramik and the Bulgarian Neolithic cultures: differences and similarities. Vegetation History and Archaeobotany. 2005;20:333–48.

Kreuz A, Marinova E. Archaeobotanical evidence of crop growing and diet within the areas of the Karanovo and the Linear Pottery Cultures: a quantitative and qualitative approach. Veget Hist Archaeobot. 2017; 26:639–57. doi: 10.1007/s00334-017-0643-x.
