# Supplementary material for: Pioneer farming in southeast Europe during the early sixth millennium BC: Climate-related adaptations in the exploitation of plants and animals
Source: PLoS One. 2018 May 18;13(5):e0197225. doi: 10.1371/journal.pone.0197225 (PMC5959071; doi:10.1371/journal.pone.0197225)
Supplement: S2 File — The file contains Table A Scores for the first six factors for Fig 1. Table B, Scores for the first six factors for Fig 3a. Table C, Scores for the first six factors for Fig 5. Table D. Scores for Fig 3b NMDS ordination based on seed counts of domestic taxa only. Plot A. Shepard plot for Fig 3b, NMDS ordination based on seed counts of domestic taxa only. Plot B, CCA ordination based on seed counts of domestic taxa. (PDF) [file pone.0197225.s002.pdf]

Table A. Scores for the first six factors for Fig. 1 (PCA of sites, altitude and bioclimatic parameters bio1, 4, 6, 9, 18; Matrix: Correlation).

	PC 1	PC 2	PC 3	PC 4	PC 5	PC 6
ACH	47.075	0.39947	-0.62415	-0.14355	0.22417	0.16191
PRO	46.696	-0.54967	-0.34468	-0.055349	0.16972	0.27333
GED	45.282	-0.6408	-0.31834	0.018923	-0.21419	0.19269
ARG	43.964	-0.59115	-0.2193	0.013298	-0.26619	0.23551
NNI	35.643	-12.725	0.38023	0.10841	-0.003714	0.14225
SES	50.859	0.39608	-0.98916	-0.1631	0.061122	0.041548
KOV	25.824	0.11037	0.49563	0.12255	0.30688	-0.097195
BAL	12.608	12.224	0.43356	0.089033	-0.0058088	-0.065903
MAD	13.389	0.029572	11.313	0.28747	-0.16193	-0.089836
CAK	15.087	0.54254	11.457	0.08744	-0.14955	-0.019606
SER	25.155	12.368	0.59569	-0.12385	-0.07573	0.052749
AZM	0.58028	-0.31137	0.79753	0.42367	-0.1348	-0.088615
YAB	14.736	-0.35074	0.57998	0.60871	-0.22235	-0.1509
ILI	21.293	0.37289	0.70251	0.1424	0.31813	-0.10934
NAB	0.92348	16.351	0.80567	0.27632	-0.3028	-0.18274
MUR	0.47589	0.90831	-0.31269	-0.94933	0.0069302	0.042845
ANZ	0.57773	0.052179	0.35662	-12.439	0.13717	0.12868
KAD	0.64883	0.27248	0.84101	0.28361	0.11359	-0.085151
KAR	0.51637	-0.098701	0.69563	0.46135	-0.23881	-0.10089
KAZ	-0.45476	0.71875	11.558	0.48891	0.041356	-0.099999
SLA	-10.864	20.112	-0.16658	-0.56701	0.16139	0.055767
BLA	-0.77639	12.631	-15.574	0.37354	-0.11849	0.006066
MIE	-33.836	13.847	0.10306	0.56052	-0.0095277	0.59842
CRI	-32.588	14.647	-0.009306	0.53935	0.032758	0.51926
CAV	-17.362	20.902	0.28037	-0.61784	0.31109	-0.0096758
GAL	-0.56445	30.387	-0.52048	-0.80865	-0.17393	-0.071581
DRE	-0.16012	0.15913	-15.442	0.27817	-0.42272	0.017578
RAK	-0.30849	42.544	0.25521	0.17338	-0.075517	-0.40092
DIV	-0.81145	10.648	-17.212	0.55625	-0.033972	0.040446
SEU	-25.593	0.58891	-0.085453	0.40572	0.071273	0.33259
FOS	-0.57548	-0.71174	-0.84819	0.15827	-0.015104	-0.022796
END	-14.862	-10.422	0.31946	-0.1627	-0.28322	0.027731

LUD	-0.81244	-0.68797	-0.43127	-0.063515	-0.08935	-0.23114
GOL	-10.422	-0.018139	-0.92275	0.27022	0.25964	-0.15171
DZH	-0.84667	-13.863	0.48508	-0.5079	0.012334	0.12498
MIH	-0.80563	-0.99186	-0.31659	-0.066343	0.23588	-0.20815
LAN	-0.91997	-0.76025	-0.50312	0.092105	0.12711	-0.018772
DON	-0.83687	-0.84488	-0.60648	0.11528	0.16723	-0.10792
KOP	-15.891	-0.6843	669	-0.42695	0.1107	0.090361
FOG	-0.59836	-0.70836	-0.82333	0.17187	-0.039413	0.0043809
OHO	-0.85035	-0.47753	16.517	0.74416	0.57857	0.062079
ORL	-19.348	0.080124	0.66917	-0.41783	0.20325	0.032325
OVC	-0.86354	-0.15323	-0.20731	-0.35212	-0.22421	-0.045252
CUI	-0.93346	-1.006	-0.39045	0.057087	0.32118	-0.14731
NOS	-0.74821	-0.58725	-0.52014	-0.061368	-0.084136	-0.24916
RÖS	-10.201	-0.83361	-0.2264	-0.06743	-0.29772	-0.058595
SAM	-12.093	-1.079	0.69143	-0.53272	0.10954	0.10465
SCH	-0.99984	-10.341	-0.25338	-4,89E-01	0.28147	-0.10294
ECS	-16.161	-10.728	0.38283	-0.15065	-0.24914	0.017904
BUC	-0.92284	-0.74561	-0.42099	0.022749	-0.28334	-0.019981
LV	-0.71207	-0.83375	-0.68271	0.10842	0.26182	-0.22036
BER	-17.587	-0.99464	0.30778	-0.051871	-0.17521	0.038671
STA	0.055552	-0.70935	-13.009	0.21946	0.44498	-0.069403
SZA	-1.374	-0.99762	0.29458	-0.22833	-0.3529	-0.015992
SZO	-13.247	-10.301	0.34334	-0.27597	-0.2928	-0.082326
TIS	-18.226	-11.506	0.58961	-0.16126	-0.17508	0.030859
ALS	-0.83573	-0.94058	-0.29251	-0.059127	0.10235	-0.051423

Table B. Scores for the first six factors for Fig. 3a (CCA ordination based on seed counts of all wild and domestic taxa).

	Axis 1	Axis 2	Axis 3	Axis 4	Axis 5	Axis 6
Cicer	2,29518	4,00863	3,75114	-2,17442	2,94434	-5,95341
Hordeum	-0,357154	0,055758	-0,692581	-0,111886	0,44725	0,520732
Lathyrus	0,732697	-0,19609	-1,38265	0,560892	0,0865459	-0,470323
Lens	0,356228	-0,201587	0,143719	-0,321281	-0,582803	0,214458
Linum	0,0701528	1,9631	1,99429	0,397045	-0,117522	3,40141
Pisum	0,535003	-0,497379	0,450246	-0,936323	0,262615	0,863401
Tr aest	0,898154	1,0855	1,77759	0,611007	0,54931	0,722854

Tr dic	0,0337071	0,0762606	-0,545292	-0,758676	0,146893	0,164265
Tr mon	-0,0603206	-0,132791	-0,211461	-0,184065	-0,0734462	-0,068262
Vicia	0,980128	1,09298	-0,292132	-1,27363	-1,5662	0,0382905
MedFruit	2,73645	-2,27589	-1,68357	1,02953	0,647849	-2,8583
Cornus mas	-0,0717129	-0,313087	0,876212	0,534914	-1,38289	-0,853325
Cornus sang	1,86494	1,78541	-3,44474	-2,3651	-0,585969	-0,115405
Corylus	0,662891	-0,0173147	-0,223125	2,96628	0,350638	-0,787113
Fragaria	-0,574278	1,0894	1,58728	0,445835	3,23742	0,728381
Malus	-0,594094	1,57119	1,67344	0,641583	1,74858	-0,319826
Physalis	-2,39456	-0,89485	-0,193578	3,61259	1,5052	-1,76133
Prunus	-0,297041	0,140873	-0,438759	1,80316	1,37882	0,909897
Quercus	0,120845	-4,75861	3,1246	-0,487682	0,490399	0,428177
Rubus	-0,150785	0,913886	0,390639	2,18569	-2,03551	0,148251
Sambucus	-2,8362	0,100951	0,777057	-0,813306	-1,47022	-1,93583
Trapa	-4,70817	-0,0908225	-0,847593	-1,20292	0,935262	-0,852249
Vitis	-0,512131	0,215045	-0,873639	0,9515	2,11176	-0,584588
ACH	0,418751	-0,752534	0,463492	0,101398	0,344808	0,0198134
ANZ	0,171812	0,173951	-0,0632111	-0,103168	0,33178	0,26714
AZM	0,28777	-0,150264	-0,511104	-0,324051	0,136393	0,126501
BAL	0,11976	-0,0334375	-0,0606715	-0,188234	0,0326773	0,237252
BER	-0,752924	0,221138	0,0978946	-0,166689	0,217876	-0,35603
BUC	-0,784257	-0,64246	0,0897335	0,500978	0,362618	-0,174139
CAV	0,179839	0,295851	0,275321	-0,0815667	-0,215704	0,367336
DRE	-0,263524	-0,102403	0,292769	-0,170544	-0,680059	-0,24808
DZH	-0,295318	-0,188733	-0,122752	0,281129	-0,0538262	0,0337528
ECS	-0,971191	0,091417	-0,211935	-0,406459	0,274844	-0,0570141
FOS	0,0229176	-1,8178	1,15761	-0,221897	-0,0854297	0,0699082
GAL	0,563777	0,809936	0,667418	-0,563803	0,545308	-0,983941
GED	0,417097	-0,0860072	-0,346979	-0,533962	-0,122593	0,0717327
ILI	0,402561	-0,33488	-0,488825	0,404164	-0,015081	-0,381084
KAD	0,115446	0,226575	-0,0553182	0,187647	0,0227455	-0,286656
KAR	0,054713	-0,0820422	-0,0798852	-0,0078497	-0,28103	-0,192364
KAZ	0,14788	0,368806	0,174018	0,0375482	-0,374621	0,263666
KOP	-0,344604	0,253079	0,21931	0,637184	-0,278586	0,33323
KOV	0,0473412	0,0450985	-0,185553	0,474813	-0,0805252	0,0165243

MUR	0,0250073	0,121514	-0,0995295	0,0411022	0,210581	0,11345
NNI	0,139052	-0,208523	-0,0382492	-0,406877	-0,148937	0,242429
OHO	-0,168236	0,0574114	-0,0131125	0,249225	-0,0143485	0,271109
ORL	-0,316342	-0,0718661	-0,146324	0,0826474	-0,0576696	-0,082416
PRO	0,18954	-0,665915	0,113051	-0,293894	6,14E-05	0,133172
SAM	-0,220653	0,0674334	-0,0777989	0,427041	0,0603431	0,0910044
SER	0,339745	0,0707744	-0,485553	0,0125439	-0,152173	-0,0312371
SES	1,18469	-1,46487	-0,449236	0,0128569	0,434806	-0,842897
SLA	0,0761322	0,274858	0,184683	0,21508	-0,024604	0,247227
TIS	-1,6485	0,0120894	-0,267784	-0,630594	0,272799	-0,286067
alt	0,39885	0,483924	0,152583	0,0592948	0,0766318	-0,0621527
bio1	0,545018	-0,48473	-0,269303	-0,107199	0,0921446	-0,0334436
bio4	-0,70995	0,351094	-0,0916662	0,24619	-0,100478	0,324053
bio6	0,629596	-0,505617	-0,212603	-0,0987288	0,108781	-0,10493
bio9	0,591883	-0,274314	-0,377693	-0,0493373	-0,0849916	-0,00410584
bio18	-0,65637	0,26896	0,3589	0,145374	-0,16617	0,0820118

Table C Scores for the first six factors for Fig. 5 (CCA ordination based on bone counts of wild and domestic taxa).

	Axis 1	Axis 2	Axis 3	Axis 4	Axis 5	Axis 6
Cattle	0,103786	-0,972431	-0,181137	-0,10691	1,65221	0,038011
Caprines	0,712188	-0,308085	1,45348	0,196814	-0,6489	-0,449076
Pig	1,7543	0,92629	-1,3525	-0,603373	-0,363498	0,256781
Sus scrofa	-1,13882	2,24157	0,879274	-0,576139	0,838624	0,264501
Bos prim	-1,31002	-0,975472	-0,422078	-1,71385	-1,26648	0,531266
Cervus	-0,894972	0,176847	-1,03746	1,29366	-0,441221	-1,46717
Capreolus	-0,362531	-0,129611	-0,116252	2,41505	-0,590611	3,27637
ACH	0,1319	0,0823016	0,0114697	-0,0294246	-0,129665	0,0190287
PRO	0,494961	-0,125095	-0,0797516	0,223952	0,0712629	-0,0625034
SES	0,430788	0,101663	-0,00680832	0,130732	0,080517	-0,0251056
ARG	0,348045	-0,002273	0,0719829	-0,14983	-0,0844571	0,037027
ANZ	0,310566	-0,0201032	0,0555758	-0,151207	-0,029864	0,0430489
KDI	0,144014	0,0148606	0,0145041	-0,0777042	0,0289941	0,0323415
CAK	0,351158	-0,0858817	-0,0614268	0,165622	0,0559829	-0,0263065
YAB	0,206063	0,04549	0,0381075	-0,0301104	0,0538755	0,0125292

KAR	-0,0222209	0,106636	-0,0562101	-0,0550911	-0,0343465	-0,00576551
MAD	0,20558	0,0454244	-0,0205152	0,0397553	0,0816323	0,000257713
NAB	0,389573	-0,173268	0,0807318	-0,135699	-0,0225689	0,0103394
MUR	0,0886033	0,192943	-0,0429594	0,0613362	0,00393165	-0,0227264
MIE	-0,103552	-0,203515	-0,0101238	-0,0239343	-0,0169712	0,0179262
DIV	-0,0222266	-0,0316518	-0,010339	-0,100475	0,00130297	0,0434921
CRI	-0,34143	-0,522003	-0,128267	0,152245	-0,015771	-0,0189103
SLA	0,0164299	0,023012	-0,0739141	-0,0666922	-0,0168415	0,0168925
RAK	-0,100815	0,0573644	-0,0936793	-0,0398263	-0,0111133	-0,00269867
BLA	-0,00876075	0,0274228	0,0219243	0,0199269	0,0273362	-0,030748
SEU	0,388104	-0,196445	-0,0559462	0,131251	0,0199138	-0,036156
GOL	-0,229765	0,093641	-0,0806598	0,0238756	-0,00840665	0,000535262
LV	-0,381416	0,0915446	-0,0746973	0,0160388	-0,00456054	-0,0131633
LUD	-0,0297616	-0,158906	0,137474	0,0135894	-0,0504438	-0,00947667
FOG	-0,0745171	0,0577486	-0,060791	-0,0742846	-0,0315986	0,00227606
KOP	-0,0724509	-0,144534	0,0512294	0,209314	0,0754951	-0,0858401
MIH	-0,167268	-0,136943	0,090178	0,0186089	-0,0154016	-0,0255471
ECS	0,0176237	-0,0678977	0,0654871	-0,112382	-0,0871315	0,0287712
DON	-0,0301695	-0,0010921	0,09997	-0,0502957	0,00403067	0,0221335
LAN	0,056007	-0,195776	0,0861526	-0,0514645	-0,0779403	0,0228306
OVC	0,0839601	-0,083305	-0,139678	0,0252693	0,02951	0,00589062
ALS	-0,188559	0,124694	-0,0369795	0,0149557	0,0130804	0,0352764
NOS	-0,254165	0,0786452	-0,0372669	-0,0761037	-0,0792099	0,0476738
RÖS	-0,214739	0,0618173	0,0190225	0,0399829	-0,0775665	-0,023324
SCH	-0,0920608	0,0441795	-0,0837191	0,0178439	-0,0272525	0,00348331
CUI	0,0136228	0,360615	-0,0526947	0,300845	0,0791849	-0,0691475
END	-0,0643986	0,0440132	0,081751	-0,100127	0,0192299	0,011217
BUC	-0,149887	-0,0731548	0,36164	0,167675	0,188753	-0,04047
STA	-0,156771	0,124461	-0,00817919	-0,0247677	0,0628065	0,0222413
SZA	-0,157431	-0,180644	0,193818	-0,0757217	0,058957	0,0351179
SZO	-0,0881926	0,0220393	0,019575	-0,0491089	-0,0240545	-0,00273152
TIS	-0,127548	0,0770038	-0,0956395	0,092189	0,00640625	-0,0120681
FOS	-0,0260351	0,0101312	-0,0312062	-0,0395265	-0,00639783	0,00690556
alt	0,160294	-0,208934	-0,31025	-0,059217	-0,0370908	0,0049236
bio1	0,625902	0,261616	0,0496793	0,0603328	0,0120168	-0,0228028
bio4	-0,421775	-0,16637	0,148588	0,0597128	0,0512506	-0,0521706

bio6	0,524349	0,409495	0,0335351	0,021942	0,00405654	-0,00127902
bio9	0,691947	0,115287	-0,0508155	-0,00422625	0,0541121	-0,00674867
bio18	-0,70192	-0,237021	-0,0693799	0,0464931	-0,0108986	-0,00770034

Name	Axis 1	Axis 2
ACH	-0,25982	-0,0031676
ANZ	-0,026623	0,048266
AZM	-0,036447	-0,0077217
BAL	-0,0051517	-0,10912
BER	0,024185	0,13406
BUC	-0,0075816	0,12778
CAV	0,31547	-0,02878
DRE	-0,040131	0,12294
DZH	-0,0056817	0,10212
ECS	0,04802	0,11579
FOS	-0,03315	0,14192
GAL	0,18262	0,09791
GED	-0,217	-0,033268
ILI	-0,09234	0,0013834
KAD	0,066537	-0,17959
KAR	0,053756	-0,095426
KAZ	0,2179	-0,095879
KOP	0,048672	0,1701
KOV	-0,080416	-0,076703
MUR	0,15563	-0,23927
NNI	-0,14426	-0,25382
OHO	0,018673	0,077978
ORL	0,056637	0,090772
PRO	-0,21529	-0,14145
SAM	0,0070724	0,10726
SER	-0,066343	-0,12261
SES	-0,2587	0,033524
SLA	0,27027	-0,1109
TIS	0,023493	0,12589

Table D. Scores for Fig. 3b (NMDS ordination based on seed counts of domestic taxa only).

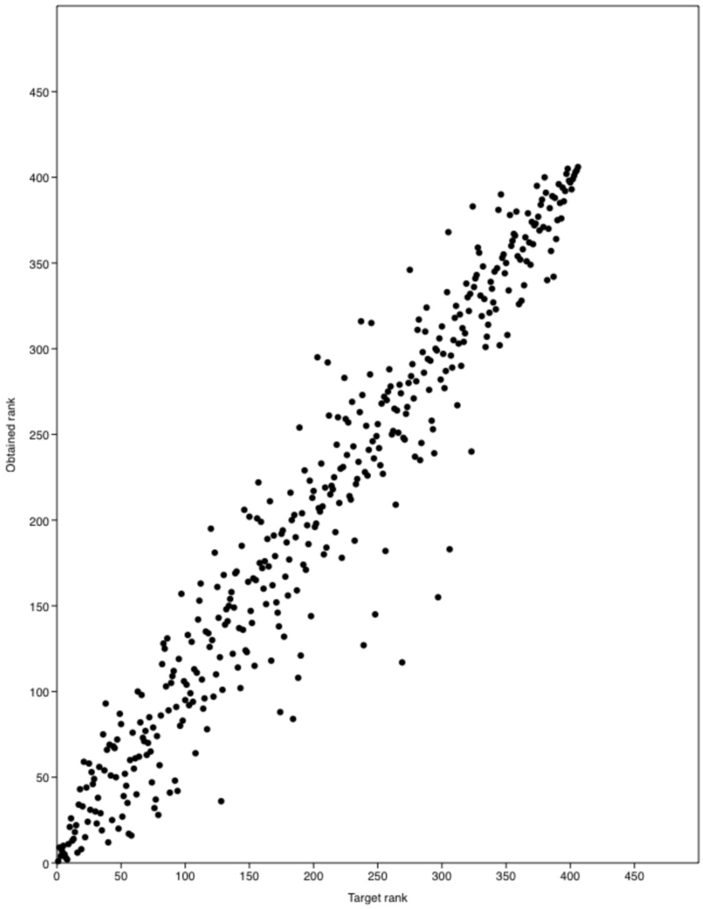

Plot A. Shepard plot for Fig. 3b (NMDS ordination based on seed counts of domestic taxa only). Stress: 0.1309.

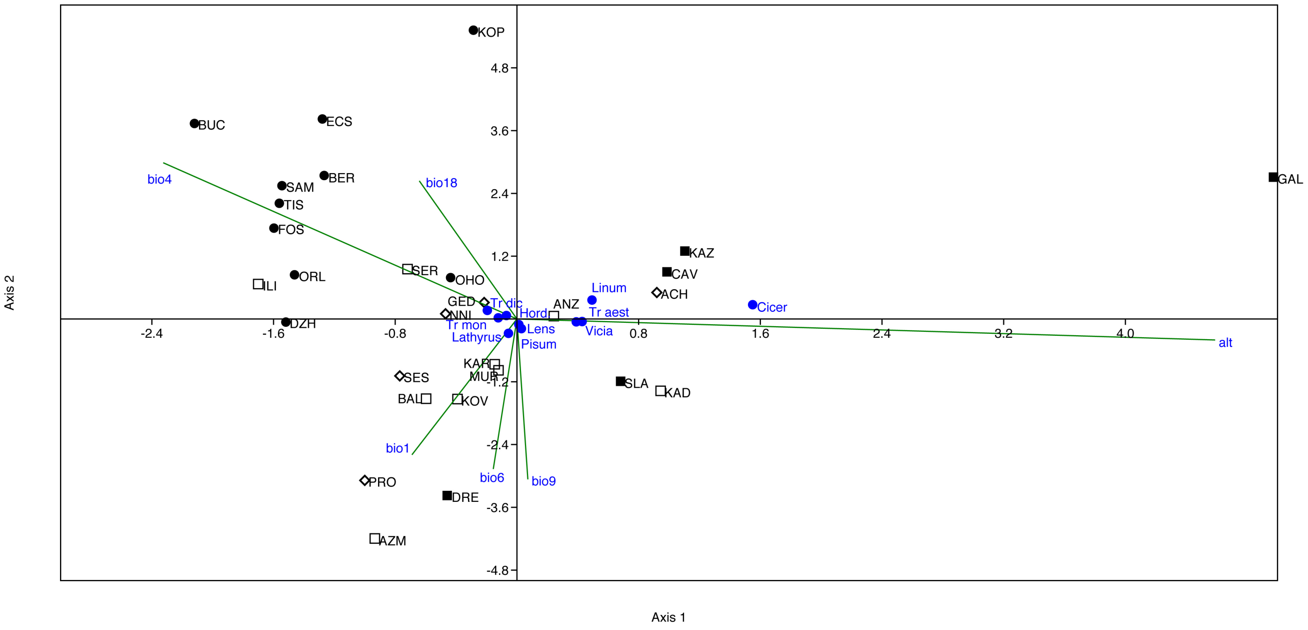

Plot B. CCA ordination based on seed counts of domestic taxa.
